# The Exocyst Subunits EqSec5 and EqSec6 Promote Powdery Mildew Fungus Growth and Pathogenicity

**DOI:** 10.3390/jof11010073

**Published:** 2025-01-17

**Authors:** Jinyao Yin, Xuehuan Zhu, Yalong Chen, Yanyang Lv, Jiaxin Shan, Yuhan Liu, Wenbo Liu, Weiguo Miao, Xiao Li

**Affiliations:** 1 Key Laboratory of Green Prevention and Control of Tropical Plant Diseases and Pests, Ministry of Education, School of Tropical Agriculture and Forestry, Haikou 570228, China; 20071000110015@hainanu.edu.cn (J.Y.); 22210904000017@hainanu.edu.cn (X.Z.); 22210904000007@hainanu.edu.cn (Y.C.); 23210904000003@hainanu.edu.cn (Y.L.); 17889980656@163.com (J.S.); 21110710000024@hainanu.edu.cn (Y.L.); saucher@hainanu.edu.cn (W.L.); 2 Danzhou Invasive Species Observation and Research Station of Hainan Province, Hainan University, Danzhou 571737, China

**Keywords:** obligate biotrophic fungi, powdery mildew, exocyst, effector protein

## Abstract

The exocyst complex in eukaryotic cells modulates secretory vesicle transportation to promote exocytosis. The exocyst is also required for the hyphal growth and pathogenic development of several filamentous phytopathogens. Obligate biotrophic powdery mildew fungi cause considerable damage to many cash crops; however, the exocyst’s roles in this group of fungi is not well studied. To verify the functions of the exocyst in powdery mildew fungus, we identified two exocyst subunits, EqSec5 and EqSec6, from *Erysiphe quercicola*, a powdery mildew fungus that infects the rubber tree *Hevea brasiliensis*. When GFP-fused EqSec5 and EqSec6 were introduced into *E. quercicola* and another phytopathogenic fungus, *Magnaporthe oryzae*, they primarily localized to the hyphal tip region. Inducing gene silencing of *EqSec5* or *EqSec6* caused growth and infection defects, and those defects could not be fully restored under the NADPH oxidase inhibitor treatment to the plant. The silenced strains also induced the host defense response including reactive oxygen species accumulation and callose deposition. The silencing of *EqSec5* or *EqSec6* also inhibited the secretion of the effector protein EqIsc1, interrupting plant salicylic acid biosynthesis. Yeast two-hybrid and gene overexpression assays suggested that EqSec5 and EqSec6 interact with each other and can complement each other’s function during host infection. Overall, our study provides evidence that the exocyst in this powdery mildew fungus facilitates effector secretion, hyphal growth, and infection.

## 1. Introduction

Exocytosis in eukaryotic cells is important for extracellular secretion and polarized membrane growth, and it is regulated by the heterooligomeric exocyst complex [1,2], which anchors secretory vesicles and facilitates the delivery and tethering of secretory vesicles to the plasma membrane [3,4,5]. The exocyst complex consists of eight subunits, such as Sec5, Sec6, and Exo70 [6,7]. The transport of proteins and lipids via exocytosis in eukaryotic cells is mediated by membrane-bound secretory vesicles [8,9]. Exocyst-mediated exocytosis is required for the pathogenicity of filamentous phytopathogens [10,11]. In the rice blast fungus *Magnaporthe oryzae*, the exocyst components assemble at the tips of growing vegetative hyphae and at the base of the appressorium penetration structure, facilitating appressorium penetration [12,13,14]. The exocyst also regulates the secretion of effectors during host colonization [15,16,17]. In a root-infecting phytopathogenic fungus, *Verticillium dahliae*, Exo70 facilitates the secretion of three small cysteine-rich proteins that contribute to virulence during root infection [18]. In the banana wilt fungal pathogen *Fusarium odoratissimum*, the exocyst subunits are localized at the tips of growing hyphae and at the outer edge of the septa in mature hyphae; Sec5 and Exo70 regulate the septum-directed secretion of a hydrolytic enzyme, α-amylase AmyB [19].

Powdery mildew fungi are a large group of obligate biotrophic parasites that infect over 10,000 species of plants, including many cash crops such as barley, wheat, cucurbits, grapevine, and rubber tree [19,20]. This group of fungi produces conidia to infect plants and develops aerial hyphae on the plant surface [20,21,22]. After penetration into plant tissues, powdery mildew fungi produce haustoria to obtain nutrients from their hosts [23,24]. These fungi were found to produce several secreted effector proteins using genomic and proteomic analyses. A portion of these effectors have no homologs in non-powdery mildew fungi [25,26,27]. The powdery mildew fungus *Erysiphe quercicola* infects the rubber tree *Hevea brasiliensis* and has become a significant limiting factor in the production of natural rubber worldwide, as natural rubber is a fundamental industrial material. The disease caused by this fungus resulted in nearly a 45% loss of natural rubber production each year in humid areas [28,29,30]. *E. quercicola* infects young tissues, such as young leaves and buds, causing leaf wilting and defoliation [31,32]. *E. quercicola* effectors, such as EqIsc1, are reported to facilitate host infection by suppressing plant SA-mediated immunity [33]. Understanding the molecular basis of pathogenicity facilitates the improvement of the strategies for plant disease management [34]. However, the mechanisms underlying the growth, pathogenic development, and effector secretion of powdery mildew fungi including *E. quercicola* remain poorly understood.

In this study, we hypothesized that the exocyst is implicated in these mechanisms. To investigate this hypothesis, we identified the exocyst subunits EqSec5 and EqSec6, which are localized in the tips of hyphae, and we found that they can affect the growth and infection of *E. quercicola*. In addition, EqSec5 and EqSec6 are associated with the secretion of effector proteins and help to suppress salicylic acid levels and plant defense responses. Further studies suggest that they interact with each other. Thus, the exocyst subunits EqSec5 and EqSec6 function together to promote pathogenicity.

## 2. Materials and Methods

### 2.1. Plant Materials and Strains

*H. brasiliensis* mildly susceptible seedlings (CATAS7-33-97) were grown in an experimental greenhouse under a 14 h light/10 h dark cycle at 25 °C (resistance genes for this plant variety have not yet been identified) [35]. The *E. quercicola* strain HO-73 obtained from Haikou, Hainan, was maintained on *H. brasiliensis* seedlings (CATAS7-33-97) with tender leaves in a growth chamber set at 25 °C with a 14 h light/10 h dark cycle. *M. oryzae* Guy11 was used as the parental wild-type strain, and it was cultured on complete medium (CM) agar plates at 28 °C in this study.

### 2.2. Sequence Analyses

Sequences were subjected to BLAST analysis at the amino acid level using the National Center of Biotechnology Information database (NCBI) (http://www.ncbi.nlm.nih.gov/, accessed on 25 May 2023). The conserved motif searches were analyzed using the MEME database (http://meme-suite.org/tools/meme, accessed on 12 June 2023). The MEGA7 software v7.0.20 was used for gene comparison and phylogenetic analysis. Phylograms were generated using the neighbor-joining method (bootstrap value: 100).

### 2.3. Gene Cloning and Plasmid Construction

The plasmids used for the electrotransformation of *E. quercicola* were constructed using the pJNARG vector as the backbone [33]. To explore the localization of EqSec5-GFP, EqSec6-GFP, *M. oryzae* Sec5 (MoSec5) (GenBank accession: MGG_07150), and *M. oryzae* Sec6 (MoSec6) (GenBank accession: MGG_03235), the constructed plasmids, expressing encoding sequences together with their native promoter, were inserted into a region containing *Kpn*I (New England BioLabs, Ipswich, MA, USA) and *Eco*RI (New England BioLabs, Ipswich, MA, USA) restriction sites in the vector using T4 ligase (Takara, Kusatsu, Japan), respectivly. To construct plasmids overexpressing *EqSec5* and *EqSec6*, the *RP27* promoter was replaced with the native promoter of the *EqSec5* and *EqSec6*, and the *EqSec5*- and *EqSec6*-encoding sequences were inserted into the *Eco*RI (New England BioLabs, Ipswich, MA, USA) restriction site using the In-Fusion^®^ Snap Assembly Master Mix (Takara, Kusatsu, Japan), respectively. To construct plasmids expressing EqIsc1-GFP (GenBank accession: QKX08450.1), the *EqIsc1*-encoding sequence, together with the native promoter, was inserted into a region containing *Kpn*I (Takara, Kusatsu, Japan) and *Eco*RI (Takara, Kusatsu, Japan) restriction sites in the vector using T4 ligase (Takara, Kusatsu, Japan). To construct the RNAi plasmids, *EqSec5*- and *EqSec6*-silencing sequences homologous to the 5’-terminal of the target gene were inserted into the *Eco*RI (Takara, Kusatsu, Japan) restriction site to ligate the RP27 promoter using the In-Fusion^®^ Snap Assembly Master Mix (Takara, Kusatsu, Japan).

### 2.4. Electrotransformation

An electrotransformation method for *E. quercicola* was implemented, as described previously [33]. Briefly, *E. quercicola* colonies, after two weeks of growth, were collected and suspended in an electroporation buffer (1 mM HEPES (pH 7.0), 50 mM mannitol, and 0.01% Tween-20). The suspension (150 μL; 1 × 10^6^ spores/mL) was mixed with 20 μg of the plasmid, kept on ice for 10 min, and then subjected to electroporation by applying two square-wave pulses of 1.7 kV for 1 ms with an interval of 5 s using an electroporation system (Bio-Rad, Hercules, CA, USA). The spores treated with electroporation were resuspended in a cold solution of 0.5 M mannitol and kept on ice for 10 min, followed by washing twice with distilled water. The washed spores were used to inoculate *H. brasiliensis* leaves. After 7 days of growth, a fluorescence microscope (Nikon Y-TV55, Minato City, Japan) was used for live-cell imaging. The hyphal tips were visualized via staining with FM4-64 (Invitrogen, Carlsbad, CA, USA) (excitation/emission: 535/610 nm). The secretion of EqIsc1 was visualized via staining with FM4-64 (Invitrogen, Carlsbad, CA, USA) (excitation/emission: 535/610 nm) and calcofluor white (CFW) (Sigma-Aldrich, St. Louis, MO, USA) (excitation/emission: 355/440 nm).

### 2.5. Silencing of Gene Expression

The spraying interference RNAs (SIGS) [36] and RNA interference (RNAi) via electrotransformation [33] were used to silence the *EqSec5* and *EqSec6* genes in *E. quercicola*. For the SIGS assay, the transcription templates of the double-stranded RNAs (dsRNAs) were amplified using the specific primer with a T7 promoter (TAATACGACTCACTATAGGG) at the 5’-end. The synthesis of the dsRNAs was performed in vitro using the T7 RNAi transcription kit (Vazyme, Nanjing, China) according to the manufacturer’s instructions. The dsRNA (20 ng/μL) was mixed with the spore suspension of *E. quercicola* (1 × 10^5^ spores/mL) and sprayed onto the surface of young *H. brasiliensis* leaves. Gene silencing was induced using electrotransformation, and plasmids carrying the *EqSec5*- and *EqSec6*-RNAi sequences were used, and the transformation method is the same as mentioned above. The transformed stains were inoculated on young *H. brasiliensis* leaves.

### 2.6. Pathogen Inoculation and Quantification

The *E. quercicola* silencing or non-silencing strains were inoculated on young *H. brasiliensis* leaves. At 7 days post-inoculation (dpi), the leaves after inoculation were inspected and photographed. In addition, the *H. brasiliensis* leaves were soaked in a decolorization solution (ethanol–acetic acid = 3:1, *v*/*v*) for 3–4 h to remove the chlorophyll. Then, the decolorized leaves were stained with an aniline blue solution (0.2 g of aniline blue (Aladdin, Shanghai, China), 1 g of K_2_HPO_4_, and 20 mL of ddH_2_O) for 30 min. The infection types of these strains were observed under a bright-field microscope channel (Nikon Y-TV55). To verify whether EqSec5 and EqSec6 were involved in plant immunity suppression, the diphenyleneiodonium (DPI) (Sigma-Aldrich, St. Louis, MO, USA) solution (10 μM) was sprayed onto the leaf surface 24 h before inoculation. The lesion areas and infection types of these strains were calculated using ImageJ v2.4.1.

### 2.7. Polyethylene Glycol (PEG)-Mediated Transformation

The *M. oryzae* protoplast preparation and fungal transformation were based on the following published protocols [37,38]. The fungal mycelium was filtered and dried through a 2-layer Miracloth. The mycelium was placed in the lysing solution (0.25 g of snailase (Yuanye, Shanghai, China), 0.25 g of lysing enzymes (Yuanye, Shanghai, China), 0.25 g of lysozyme (Chinken egg) (Yuanye, Shanghai, China), 0.15 g Driselase^TM^ Basidiomycetes sp (Merck KGaA, Taufkirchen, Germany)., add 0.7 M NaCl solution to 25 mL) and cultured at 28 °C for 1.5–2 h. Finally, the concentration of the released protoplasts was adjusted to 1 × 10^8^ cells/mL for transformation. The protoplasts were mixed with the EqSec5-pJNARG and EqSec6-pJNARG plasmids (2 μg), respectively. The mixtures were maintained for 25 min at room temperature, and 1 mL of PTC solution (60% PEG4000, 20% *w*/*v* sucrose, 50 mM Tris-HCl (pH = 8.0), and 50 mM CaCl_2_) was added to incubate the mixtures at room temperature for 25 min. The protoplast mixtures were added to TB3 solid medium plus 50 μg/mL of ampicillin and 50 μg/mL of geneticin (G418) [39], cultured at 28 °C for 1.5–2 h, and then overlaid and cultured with another molten TB3 solid medium plus 5 μg/mL of ampicillin and 100 μg/mL of G418 in dark conditions at 28 °C for 7–10 days. Potential fungal transformants were subcultured on CM solid medium (100 μg/mL of G418) and confirmed via PCR screening. The transformants were collected and shaken for 24 h in CM containing G418. A fluorescence microscope (Nikon Y-TV55) was used for live-cell imaging. The hyphal tips were visualized via staining with FM4-64 (excitation/emission: 535/610 nm).

### 2.8. qRT-PCR and RT-PCR

The *E. quercicola* strains were inoculated on *H. brasiliensis* leaves, and *M. oryzae* strains were cultured on CM plates for 7 days; RNA samples were isolated using the Fungal RNA Extraction Kit (OMEGA, Biel/Bienne, Switzerland). The cDNA was synthesized using the PrimeScript^TM^ II 1st Strand cDNA Synthesis Kit (TaKaRa, Kusatsu, Japan). Quantitative RT-PCR (qRT-PCR) was performed using the QuantStudio^TM^ 5 real-time PCR instrument (Thermo Fisher Scientific, Waltham, MA, USA). Gene expression levels were normalized to *EqEF-1a* transcript levels in *E. quercicola* according to the ΔΔCt method. For reversed transcript PCR (RT-PCR) [40], *EqEF-1a* and *β-actin* were used as the reference genes in *E. quercicola* and *M. oryzae*, respectively. The primer pairs are listed in Table S1. The PCR conditions were as follows: 95 °C for 5 min followed by 30 cycles of 95 °C for 30 s, 58 °C for 30 s, and 72 °C for 30 s.

### 2.9. Histochemical Staining and Quantification of Plant Defense Responses

For the visualization of callose, the *H. brasiliensis* leaves infected with *E. quercicola* were treated with a decolorization solution (ethanol–acetic acid = 3:1, *v*/*v*) for 4 h to remove the chlorophyll. Then, the decolorized leaf disks (6 mm in diameter) were treated with an aniline blue solution (0.2 g of aniline blue (Aladdin, Shanghai, China), 1 g of K_2_HPO_4_, and 20 mL of ddH_2_O) for 2–4 h in the dark at room temperature. Callose deposition was analyzed under a fluorescence microscope using the DAPI channel (emission/excitation: 488 nm/340 nm) (Nikon Y-TV55) [41,42].

The level of ROS accumulation was measured using the DAB (Aladdin, Shanghai, China) staining and DCFH-DA methods (Geruisi, Suzhou, China) [43]. For the DAB staining of *H. brasiliensis* leaves infected with *E. quercicola*, leaf disks (8 mm in diameter) were soaked in 20 mL of 0.1% DAB (Aladdin, Shanghai, China) solution overnight, and 95% ethanol was used to remove the chlorophyll. The samples were then analyzed under a microscope (Nikon Y-TV55). DCFH-DA (Geruisi, Suzhou, China), an oxidation-sensitive probe, can be converted into a substance that can generate fluorescent signals, namely, dichlorofluorescein (2′,7′-dichlorofluorescein (DCF)). This fluorescence signal can be examined under a microplate reader (Tecan Infinite E Plex, Männedorf, Switzerland); excitation/emission: 488/525 nm), and its fluorescence intensity is proportional to the level of reactive oxygen species. The measurement of ROS was performed according to the manufacturer’s instructions (Grace biotechnology, G0163W, Bend, OR, USA). The leaf disks (8 mm in diameter) from the *H. brasiliensis* leaves infected with *E. quercicola* were incubated with DCFH-DA (Geruisi, Suzhou, China) for 30 min at 37 °C in the dark. The following fluorescent value was measured with the microplate reader. The ROS intensity content in the leaf tissues was calculated according to the measured standard curve (the ROS intensity = fluorescent value/T/W).

### 2.10. Measurement of SA Concentrations

Free salicylic acid (SA) extracted from *H. brasiliensis* leaves was quantified using LC-MS, as previously described [44]. Briefly, leaf powder from each sample (0.3 g) was ground in liquid nitrogen. The supernatant containing the free SA and SA conjugates was extracted and filtered; the filtered solution was loaded into an LC-MS platform (Agilent Technologies Inc., Santa Clara, CA, USA) for free SA measurement. The SA content in the leaf tissues was calculated according to the measured standard curve (Y = 0.1574 x + 0.0773; R2 = 0.9974).

### 2.11. Yeast Two-Hybrid Assays

Yeast two-hybrid (Y2H) assays were performed using the GAL4 system. The full-length coding sequences of *EqSec5* were amplified and cloned into a pGBKT7 vector with *Bam*HI (Takara, Kusatsu, Japan); moreover, the complete sequences of *EqSec6* were cloned into pGADT7 with *Bam*HI (Takara, Kusatsu, Japan). Empty pGADT7 and pGBKT7 vectors were used as negative controls. The vectors were transformed into the yeast strain Y2H using the lithium acetate method [45]. The protein–protein interaction was detected on SD/−Leu/−Trp/−His/−Ade + X-α-gal plates.

### 2.12. Statistical Analysis

Student’s *t*-tests were performed to compare the means between two data groups. One-way analysis of variance and Tukey’s test were used to compare multiple data groups. *p* < 0.01 or 0.05 represented a significant difference.

## 3. Results

### 3.1. Identify the Homologs of Sec5 and Sec6 in E. quercicola

The *E. quercicola* genome encodes six homologs of exocyst subunits, including EqSec3, EqSec5, EqSec6, EqSec8, Exo70, and Exo84. Total RNA was extracted from colonies with 7 days of growth, and RT-PCR was conducted to amplify the coding sequences of these six proteins. The results show that the bands of *EqSec5* and *EqSec6* were stronger, and those of *EqSec3*, *EqSec8*, *Exo70,* and *Exo84* were weak or absent, likely due to the higher biomass of *EqSec5* and *EqSec6* (Figure S1). We found that EqSec5/6 displayed high amino acid similarities (>50%) with the reported Sec5/6 from *Erysiphe necator*, *M. oryzae*, *Aspergillus nidulans*, and *F. odoratissimum* using NCBI BlastP tools. A phylogenetic tree was constructed using the neighbor-joining (NJ) method, which showed that EqSec5 and EqSec6 are more similar to another Sec5/6 from *Erysiphe* (Figure S2). The MEME online software (http://meme-suite.org/tools/meme, accessed on 12 June 2023) combined with the visualized motif pattern function of TBtools was used to analyze the sequence diversity of the Sec5 and Sec6 sequences (Figure S2). The results show that both EqSec5/6 had 12 motifs, similar to the Sec5 and Sec6 identified in other fungi.

### 3.2. EqSec5 and EqSec6 Localize to the Hyphal Tips

**Figure 1:**
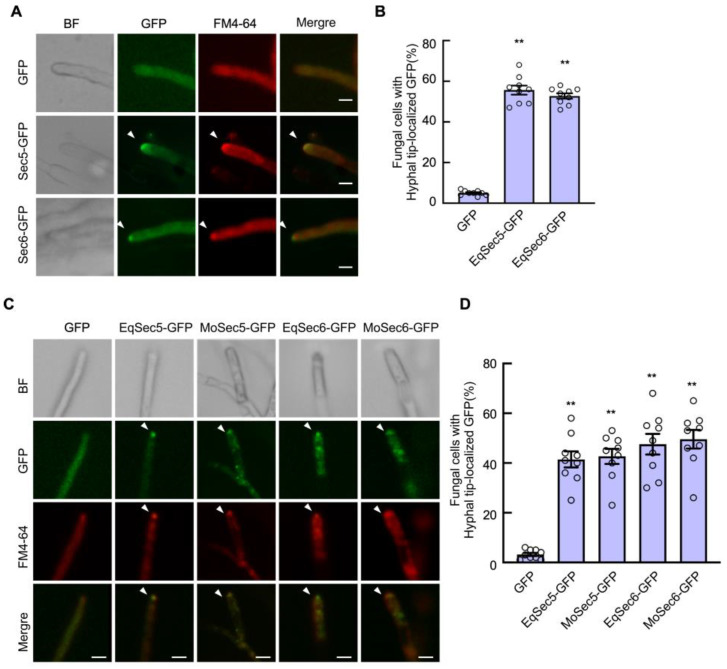
Subcellular localizations of EqSec5- and EqSec6-GFP in hyphae of *E. quercicola* and *M. oryzae*. (**A**) EqSec5- and EqSec6-GFP in hyphal tip region of *E. quercicola*. Arrows indicate the hyphal tip-localized region. FM4-64 was used to label the membrane and the Spitzenkörper. Bars: 50 μm. (**B**) Quantification of fungal cells with hyphal tip-localized GFP of *E. quercicola*. (**C**) EqSec5-GFP, EqSec6-GFP, MoSec5-GFP, and MoSec6-GFP in hyphal tip region of *M. oryzae*. Arrows indicate the hyphal tip-localized region. FM4-64 was used to label the membrane and the Spitzenkörper. Bars: 50 μm. (**D**) Quantification of fungal cells with hyphal tip-localized GFP of *M. oryzae*. In (**B**,**D**), the data represent means ± SE (n = 9 replicates from 3 independent experiments). The GFP stain was used as the control. Significant differences between the two data groups were analyzed using Student’s *t*-test (** *p* < 0.01).

We studied the subcellular locations of EqSec5 and EqSec6 to ensure that they were exocyst subunits. We constructed a vector designed to express GFP-fused EqSec5 or EqSec6 under the control of their native promoters and introduced these vectors into *E. quercicola* conidia using the electrotransformation method. The conidia were allowed to grow on *H. brasiliensis* leaves treated with carbendazim for transformant selection. These transformants were validated via RT-PCR (Figure S3A) and then subjected to fluorescence microscopic analysis at 7 dpi. We found that both EqSec5- and EqSec6-GFP formed spherical structures at the apex of aerial hyphae, and they were colocalized with or near the Spitzenkörper, which were labeled with the membrane-stained marker FM4-64 (Figure 1A,B). GFP alone expressed in *E. quercicola* was used as the control; it was primarily distributed in the cytoplasm. To further verify the localization of EqSec5 and EqSec6, MoSec5 and MoSec6 were selected as positive controls, and these GFP-fused proteins were introduced into *M. oryzae* strain Guy11 via PEG-mediated transformation (Figure S3B). As expected, all the exocyst subunit proteins were distributed in the hyphal tip areas and colocalized with or near the Spitzenkörper (Figure 1C,D). These results show that EqSec5 and EqSec6 are exocyst components.

### 3.3. EqSec5 and EqSec6 Promote E. quercicola Pathogenesis and Growth

**Figure 2:**
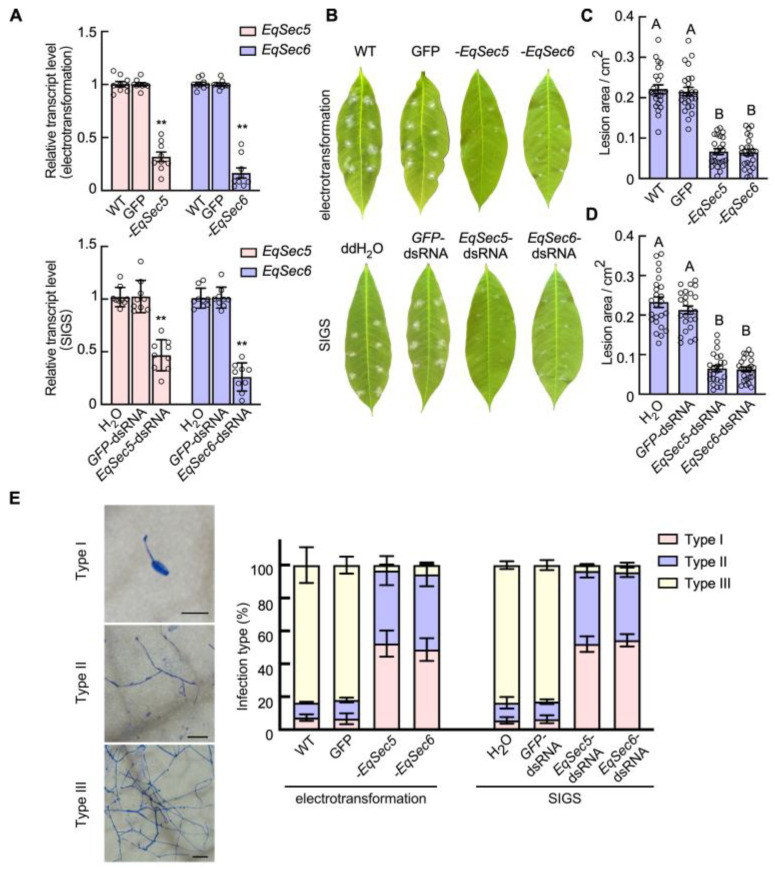
The silencing of *EqSec5* and *EqSec6* inhibited the pathogenicity of *E. quercicola*. The gene silencing of *EqSec5* and *EqSec6* was induced via electrotransformation and a dsRNA treatment. (**A**) Relative transcript levels of *EqSec5* and *EqSec6* were determined using qRT-PCR. The data represent means ± SE (n = 9 replicates from 3 independent experiments). The levels of *EqSec5* and *EqSec6* in the wild-type (WT) or H_2_O-treated strain were normalized to 1.0. Student’s *t*-test was used to analyze significant differences (** *p* < 0.01). (**B**) Disease symptoms on *H. brasiliensis* leaves inoculated with *E. quercicola* stains. Representative images were captured at 7 dpi. (**C**) Quantification of lesion area in *H. brasiliensis* leaves inoculated with *E. quercicola* strains via electrotransformation at 7 dpi. (**D**) Quantification of lesion area in *H. brasiliensis* leaves inoculated with *E. quercicola* strains via SIGS at 7 dpi. In (**C**,**D**), significant differences are indicated by different letters according to one-way analysis of variance and Turkey’s multiple-comparison test (*p* < 0.01), and the data represent means ± SE (n = 24 inoculated sites from 3 independent experiments). (**E**) The quantification of infection types for *E. quercicola* strains inoculated onto *H. brasiliensis* leaves at 7 dpi. The infection of each conidium was divided into three types: no penetration (type I), limited hyphal growth (colony radius < 150 μm; type II), and extended hyphae (colony radius > 150 μm; type III). Leaf tissues were bleached, and hyphae were stained with aniline blue. The experiments were conducted three times with similar results. Bars: 20 μm. In (**A**–**E**), *-EqSec5*/*6*—the strains transformed with *EqSec5/6*-silencing plasmids; GFP—the strain transformed with GFP.

We attempted to investigate the roles of EqSec5 and EqSec6 in pathogenicity and growth. We induced gene silencing using two methods, SIGS and electransformation with RNAi plasmids, to investigate the roles of EqSec5 and EqSec6 in *E. quercicola* pathogenicity. These dsRNAs were homologous to the 1–250 bp coding sequences. Using NCBI BLAST analysis, we found that the sequences used for gene silencing could not match any other sequences longer than 20 bp in *E. quercicola*. A qRT-PCR analysis revealed that the application of both methods reduced the transcript levels of *EqSec5* or *EqSec6* by almost 50% at 7 dpi (Figure 2A). At 7 dpi, the lesion area of *EqSec5*- and *EqSec6*-silenced strains generated using two silenced methods were reduced by more than 70% (Figure 2B–D). The strains without gene silencing developed dense colonies with extended hyphae. In contrast, the growth of both *EqSec5*- and *EqSec6*-silenced strains was inhibited. Meanwhile, we observed that the silencing of *EqSec5* and *EqSec6* led to a large proportion of conidia with no germination or penetration (Figure 2E). Only a small portion of the *EqSec5-* and *EqSec6*-silenced strains (less than 2%) penetrated successfully, but they were inhibited from extending hyphae (Figure 2E). These results suggest that both EqSec5 and EqSec6 promote fungal penetration and growth.

To investigate whether the reduction in the gene-silenced strains in pathogenicity and growth was associated with host defense, we added the NADPH oxidase inhibitor DPI to suppress plant responses in the *H. brasiliensis* leaves, followed by the inoculation with the *EqSec5*- and *EqSec6*-silenced strains, which were generated via a dsRNA treatment. DPI application increased the infection rates of these gene-silenced strains but did not fully restore them to the levels of the strains without gene silencing, suggesting that EqSec5 and EqSec6 are involved in both fungal growth and plant immunity suppression (Figure S4). However, we did not observe curved or zigzag-shaped hyphae from the hyphae developed with these gene-silenced strains (Figure S5). The presence of curved or zigzag-shaped hyphae indicates defects in maintaining cell polarity [46].

### 3.4. EqSec5 and EqSec6 Regulate Secretion of the Effector EqIsc1

**Figure 3:**
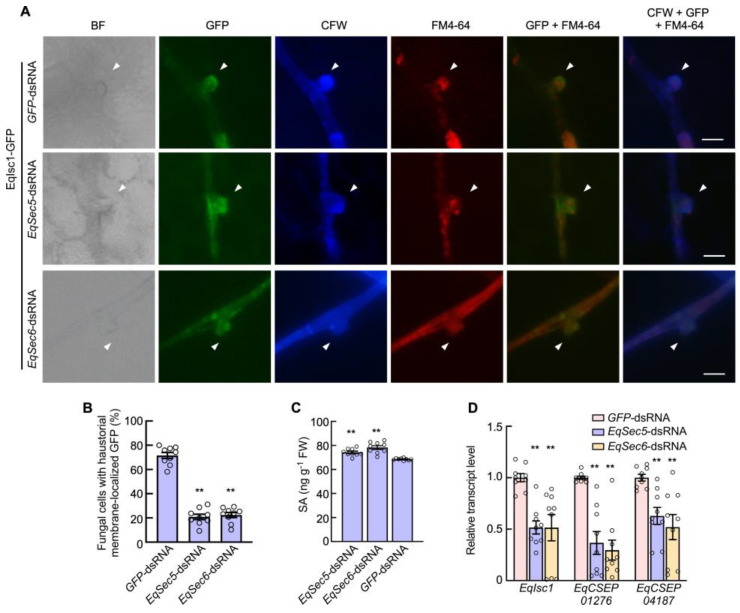
EqSec5 and EqSec6 regulate the secretion of effector proteins. (**A**) The localization of EqIsc1-GFP in hyphae and haustoria of *E. quercicola* with dsRNA treatment. Arrows indicate the haustoria. The dyes CFW and FM4-64 were used to label the cell wall and membrane. Bars: 200 μm. (**B**) The percentage of haustoria with EqIsc1-GFP localized on the periphery of cells. The GFP strain was used as the control. (**C**) Relative transcript levels of *EqIsc1*, *EqCSEP01276,* and *EqCSEP04187* were determined using qRT-PCR. The level of each gene in the strain with the GFP-dsRNA treatment was normalized to 1.0. (**D**) Free SA levels in *H. brasiliensis* leaves infected with *E. quercicola* strains. The GFP strain was used as the control. In (**B**–**D**), the data represent means ± SE (n = 9 inoculated sites from 3 independent experiments). Significant differences between the two data groups were analyzed using Student’s *t*-test (** *p* < 0.01).

We investigated whether exocyst subunits are associated with effector secretion; a reported effector, EqIsc1, was tested. We introduced GFP-fused EqIsc1 into *E. quercicola* using electrotransformation, and the transformed strain was then exogenously treated with dsRNAs designed to induce *EqSec5* or *EqSec6* silencing. The transformants were validated via RT-PCR (Figure S3C). A strain without the dsRNA treatment was used as the control, and it showed that EqIsc1-GFP was localized in the periphery of the haustoria, where it was labeled with calcofluor white (the fungal cell wall marker) and FM4-64 (membrane staining), as previously described [33]. This localization pattern implied that EqIsc1 was delivered into the extra-haustorial membrane, the interface between the plant and fungus. In contrast, in the silenced strains, EqIsc1-GFP was primarily localized in the cytoplasm of the haustoria, suggesting that the transport of EqIsc1 to the plasma membrane was inhibited by the silencing of *EqSec5* or *EqSec6* (Figure 3A,B). Moreover, a qRT-PCR analysis revealed that *EqSec5* or *EqSec6* silencing reduced the transcript levels of effector genes, including *EqIsc1*, *EqCSEP01276*, and *EqCSEP04187* (Figure 3C). Based on these results, we conclude that EqIsc1 secretion is regulated by these two exocyst subunits.

### 3.5. EqSec5 and EqSec6 Silencing Induced the Host Defense Response

EqIsc1 can function to suppress SA biosynthesis in *H. brasiliensis* [33]. We suspected that the EqIsc1 secretion defect caused by *EqSec5* and *EqSec6* silencing may affect the SA level in the plant. To test this, we allowed the *EqSec5*- and *EqSec6*-silenced strains to infect *H. brasiliensis*, and the SA level in the leaves was measured using the HPLC method. The *EqSec5*- and *EqSec6*-silenced strains significantly induced the SA level compared with the controls, the strain without treatment or treated with GFP-dsRNA (Figure 3D).

**Figure 4:**
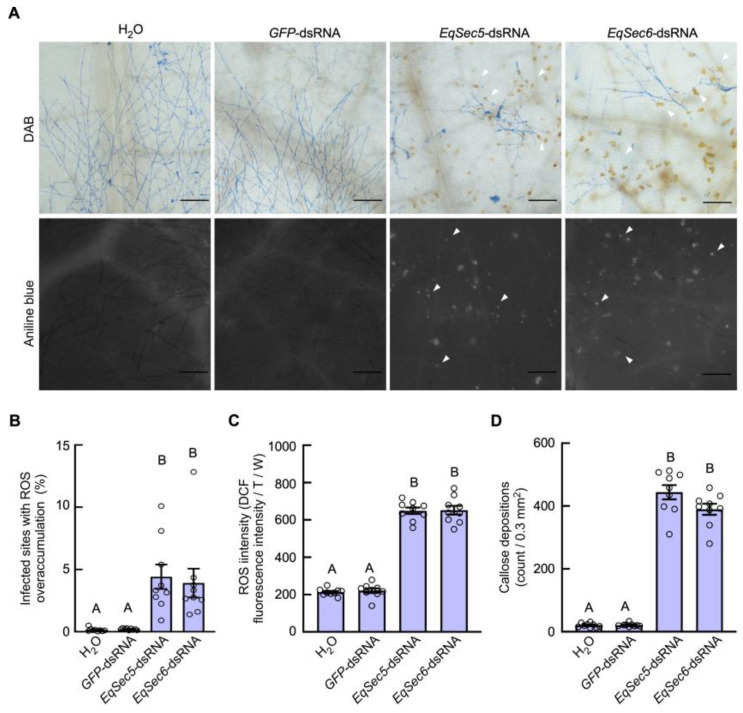
*EqSec5* and *EqSec6* silencing elicited defense responses in *H. brasiliensis*. (**A**) Callose deposition and ROS accumulation in infected leaves (7 dpi) were detected with aniline blue and DAB staining. Bars: 200 μm. Arrows indicate the ROS accumulation and callose deposition, respectively. (**B**) Quantification of the infected sites (7 dpi) with ROS accumulation labeled via DAB staining. The percentage of ROS staining area/0.3 mm^2^ leaf area was calculated. (**C**) The determination of ROS levels in infected leaves (7 dpi) using DCFH-DA. (**D**) Quantification of the infected sites with callose deposition. In (**B**–**D**), the data represent means ± SE (n = 9 replicates from 3 independent experiments). Significant differences are indicated by different letters according to one-way analysis of variance and Turkey’s multiple-comparison test (*p* < 0.01).

Furthermore, we tested the plant defense responses, including ROS accumulation and callose deposition, in the leaves infected by the gene-silenced strains. ROS accumulation was detected using DAB and DCFH-DA. The DAB-stained areas in leaves infected with the *EqSec5*- or *EqSec6*-silenced strain were significantly larger than those infected by the control strains (Figure 4A,B). We also found that these gene-silenced strains induced higher ROS levels using a microplate reader (Tecan Infinite E Plex), which detected the signals of DCFH-DA-labeled ROS (Figure 4C). Similarly, we detected more areas with callose deposition in the leaves infected by these gene-silenced strains compared with those infected by the controls (Figure 4A,D). Thus, EqSec5 and EqSec6 facilitate the fungus to suppress host defense responses.

### 3.6. EqSec5 and EqSec6 Function Together to Regulate Pathogenicity

**Figure 5:**
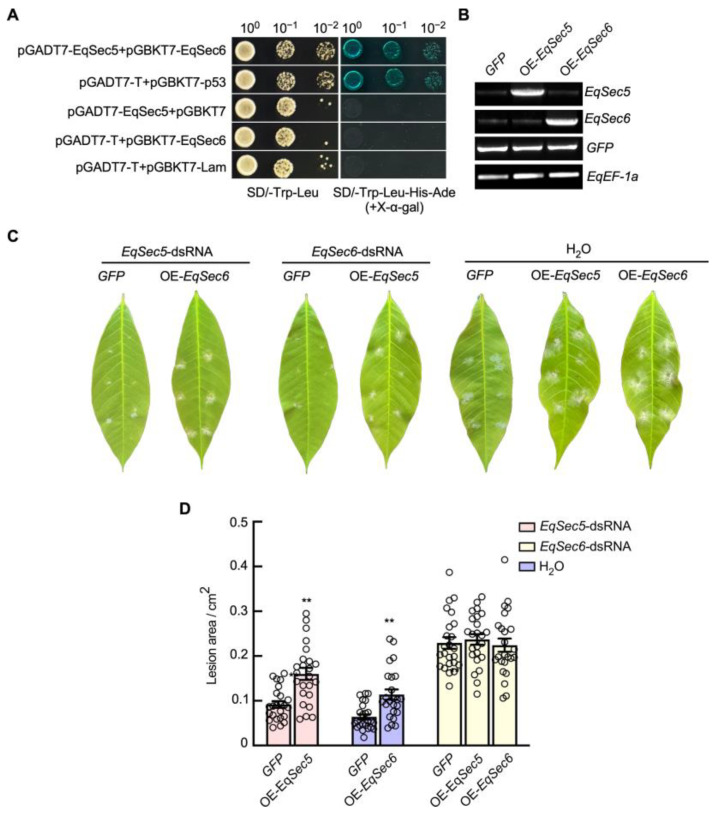
EqSec5 and EqSec6 function together. (**A**) Y2H assay analyzing the interaction between EqSec5 and EqSec6. EqSec5 proteins were fused with the GAL4 activation domain (AD) and EqSec6 proteins with the binding domain (BD). The empty pGADT7 and pGBKT7 vectors were used as negative controls. Yeast cells containing the indicated plasmids were plated on growth medium (–Leu/–Trp) or selection medium (–Leu–Trp–His–Ade) supplemented with X-α-gal. (**B**) RT-PCR analysis for *EqSec5/6* overexpression. RNA samples were extracted from strains with 7 days of growth. *EqEF-1a* was used as the reference control. (**C**) Overexpressing *EqSec5* and *EqSec6* partially restored infection caused by the *EqSec6*- and *EqSec5*-silenced strains, respectively. The representative images were captured at 7 dpi. (**D**) Quantification of lesion area in *H. brasiliensis* leaves inoculated with *E. quercicola* strains at 7 dpi. The data represent means ± SE (n = 24 inoculated sites from 3 independent experiments). Student’s *t*-test was used to analyze significant differences between the two data groups (** *p* < 0.01). In (**B**–**D**), OE-*EqSec5/6* is the strain overexpressing *EqSec5/6*.

Given that both *EqSec5* and *EqSec6* silencing affected *E. quercicola* pathogenicity, we next investigated the roles of these two subunits in fungal invasion. Firstly, the Y2H assay was carried out with EqSec5 as a bait and EqSec6 as a prey. The yeast cells transformed with both BD-EqSec5 and AD-EqSec6 grew on SD medium without Leu, Trp, His, and Ade, suggesting an interaction between the two proteins (Figure 5A). Next, using a constitutive promoter, *RP27*, to overexpress *EqSec5* in the *EqSec6*-silenced strain increased its infection rate. Similar results were observed by overexpressing *EqSec6* in the *EqSec5*-silenced strain (Figure 5B–D). Thus, EqSec5 and EqSec6 have redundant functions in infection.

## 4. Discussion

In this study, we identified the homologs of exocyst subunits Sec5 and Sec6 in *E. quercicola*. In the phylogenetic tree and motif analyses, Sec5 and Sec6 in *E. quercicola* contained Sec5 and Sec6 conserved motif similarities with other characterized exocyst subunits, respectively. We transformed EqSec5- and EqSec6-GFP into *E. quercicola* and *M. oryzae* and observed the localizations of these two fusion proteins in the hyphal tip regions. The localization patterns of EqSec5 and EqSec6 were similar to those of exocyst proteins in *F. odoratissimum* and *M. oryzae* [19,38]. Exocyst subunits can reach the cell poles via actin and microtubule cytoskeleton-dependent transport [47,48]. During hyphal tip growth, secretory vesicles accumulate in the Spitzenkörper (apical body), also known as the vesicle supply center, which regulates polarized growth [22,49,50]. In our study, we found that the localization patterns of EqSec5 and EqSec6 are not entirely consistent with the Spitzenkörper, which are similar to the localization of the exocyst in *F. odoratissimum* and *M. oryzae* [19,38]. It is known that the assembly of exocyst subunits in the hyphal tip region is corelated with hyphal growth rate [51], and exocytosis is thought to occur at the hyphal tip regions in filamentous fungi, where enzymes and other proteins can be secreted through this process [52]. Therefore, the localizations of EqSec5 and EqSec6 suggest their roles in hyphal growth.

It has been reported that the exocyst regulates pathogenicity in several phytopathogenic fungi [53,54]. Here, we showed that *EqSec5* and *EqSec6* silencing significantly reduced the conidial penetration, hyphal development, and lesion areas of *E. quercicola*, suggesting that exocyst components contribute to pathogenicity. *M. oryzae* exocyst subunits such as Exo70, Sec5, and Sec6 assemble at the base of the appressorium before infection [54]. Exocyst organization in the appressorium is regulated by septin GTPase Sep3, MAPK pmk1, and NoxR-Nox2 NADPH oxidase [55]. Mutations in Exo70, Sec5, and Sec6 reduced infection ability [56]. Additionally, *M. oryzae* conidia secrete mucilage to attach to leaves. This secretion was affected by Exo70 and Sec5 mutations [38]. We found that the *EqSec5*- and *EqSec6*-silenced strains produced more unsuccessfully penetrated conidia than the controls; therefore, *EqSec5* and *EqSec6* silencing may affect the secretion required for conidia to attach to leaves and normal appressorium differentiation.

We also found that DPI treatment, which is supposed to inhibit plant ROS-mediated basal defense, did not fully restore infection caused by the *EqSec5*- and *EqSec6*-silenced strains at 7 dpi, implying that EqSec5 and EqSec6 have roles in hyphal cell growth. The plant defense response was enhanced in *H. brasiliensis* leaves inoculated with the *EqSec5*- or *EqSec6*-silenced strains, suggesting that EqSec5 and EqSec6 are required for the pathogen’s ability to suppress host immunity. However, we did not observe a deficiency of cell polarity in the hyphae of the *EqSec5*- and *EqSec6*-silenced strains. Similarly, mutations in *M. oryzae* Sec6 and *F. odoratissimum* Exo70 or Sec5, which reduced the growth rates of aerial hyphae on artificial culture media, did not alter cell polarity [19,38]. In contrast, a mutation in *A. nidulans* TeaA, the cell-end marker protein that promotes actin organization, resulted in zigzag-shaped hyphae, indicating a lack of hyphal polarity [57]. Thus, the exocyst is not essential for polarity establishment in fungi.

It is well known that the secretion of effector proteins is the key factor affecting the efficient infection of many pathogenic fungi [58,59]. Previous studies have shown that *E. quercicola* can secret effectors to establish a successful interaction with its host [35], but the secretory mechanisms are not known. Our results suggest that EqIsc1 effector secretion by *E. quercicola* was affected by *EqSec5* and *EqSec6* silencing. EqIsc1 localization in the periphery of the haustoria partially disappeared in the infection sites with the gene-silenced strains, probably due to a delay or an inhibition of EqIsc1 exportation. Additionally, the SA level was elevated in the leaves inoculated with the gene-silenced strains; thus, the defect of effector exportation prevented EqIsc1 from suppressing SA biosynthesis. Moreover, we note that EqSec5 and EqSec6 may regulate the secretion of various effectors. Several immune signaling pathways in addition to the SA pathway were possibly impacted by *EqSec5* and *EqSec6* silencing. The identification of all effectors regulated by the exocyst will be an interesting topic for future investigation.

Our findings suggest that EqSec5 and EqSec6 exhibit redundant functions in the pathogenicity of *E. quercicola*. The Y2H assay indicated an interaction between EqSec5 and EqSec6. Notably, the infection rate of the *EqSec6*-silenced strain increased upon overexpression of EqSec5, while the infection rate of the *EqSec5*-silenced strain also increased when EqSec6 was overexpressed. As mentioned above, EqSec5 and EqSec6 display a similar localization pattern in the hyphal tip region, and both are implicated in the regulation and secretion of the EqIsc1 effector. We propose that EqSec5 and EqSec6 may partially compensate for one another in some cases during the exocytosis process. Furthermore, the redundancy observed between EqSec5 and EqSec6 is likely a result of evolutionary processes that enhance exocytosis in pathogenic fungi, thereby increasing their adaptability to host plants.

In the yeast *Saccharomyces cerevisiae*, exocyst subunits interact with Rho GTPases including Rho1, Cdc42, and Rho3, as well as the t-SNARE proteins Sso1/2 and Sec9, the v-SNARE proteins Snc1/2, and the phospholipid phosphatidylinositol 4,5-bisphosphate [PI(4,5)IP2] [15,60]. These interactions are critical for SNARE (N-ethylmaleimide-sensitive factor attachment protein receptors) assembly, which in turn facilitates secretory vesicle fusion with the target sites in the plasma membrane [61]. Additionally, Sec5 and Sec6 form a subcomplex of the exocyst [6]. Sec5 coordinates the interaction among subunits, while Sec6 interacts with SNARE proteins [15,62]. In phytopathogenic fungi, although exocyst components potentially employ conserved mechanisms to function in exocytosis, it additionally facilitates the development of pathogenic structures such as appressoria, infection hyphae, and haustoria, as well as the secretion of virulence factors such as effectors [63,64]. Moreover, the studies of the exocyst in phytopathogenic fungi have practical implications, as those results suggest the potential strategy of developing fungicides that specifically target this structure.

Taken together, we characterize two exocyst subunits Sec5 and Sec6 from the powdery mildew fungus *E. quercicola*. Both subunits have important roles in fungal growth and effector secretion, thereby contributes to pathogen infection and the suppression of host defense response.

## 5. Conclusions

Two homologs of exocyst subunits, EqSec5 and EqSec6, were identified from *E. quercicola*. In the hyphal cells, the EqSec5- and EqSec6-GFP structures were localized to the hyphal tip regions. The silencing of *EqSec5* and *EqSec6* resulted in the inhibition of growth, infection, and effector secretion in *E. quercicola*. The silencing also prevented the fungus from suppressing SA production and defense response in the host plant. The Y2H assay indicated that EqSec5 and EqSec6 interact with each other, while the gene overexpression assay suggests their redundant functions during infection. Collectively, EqSec5 and EqSec6 play significant roles in fungal growth and pathogenicity.

## Data Availability

The gene sequences used in this study are available from the NCBI database (https://www.ncbi.nlm.nih.gov/, accessed on 7 September 2023). The *E. quercicola* genome sequences have been deposited in GenBank under the accession codes of GCA_003957845.1.

## Supplementary Materials

The following supporting information can be downloaded at the following website: https://www.mdpi.com/article/10.3390/jof11010073/s1, Figure S1: RT-PCR analysis of exocyst subunits using total RNA from *E. quercicola* as a template. *EqEF-1a* was used as the reference control; Figure S2: Phylogenetic trees and conservative protein motifs of EqSec5 and EqSec6; Figure S3: RT-PCR analysis of transgenes in *E. quercicola* and *M. oryzae* transformants; Figure S4: DPI treatment did not fully restore infection caused by the *EqSec5*- and *EqSec6*-silenced strains; Figure S5: The EqSec5- and EqSec6-silenced strains did not exhibit hyphal morphological defects, such as curved or zigzag-shaped hyphae.; Table S1: Primers used in this study; Table S2: DNA sequences used in this study.
