# Supplementary material for: The Exocyst Subunits EqSec5 and EqSec6 Promote Powdery Mildew Fungus Growth and Pathogenicity
Source: J Fungi (Basel). 2025 Jan 17;11(1):73. doi: 10.3390/jof11010073 (PMC11767214; doi:10.3390/jof11010073)
Supplement: Supplementary file 1 [file jof-11-00073-s001.zip › Figure S1.pdf]

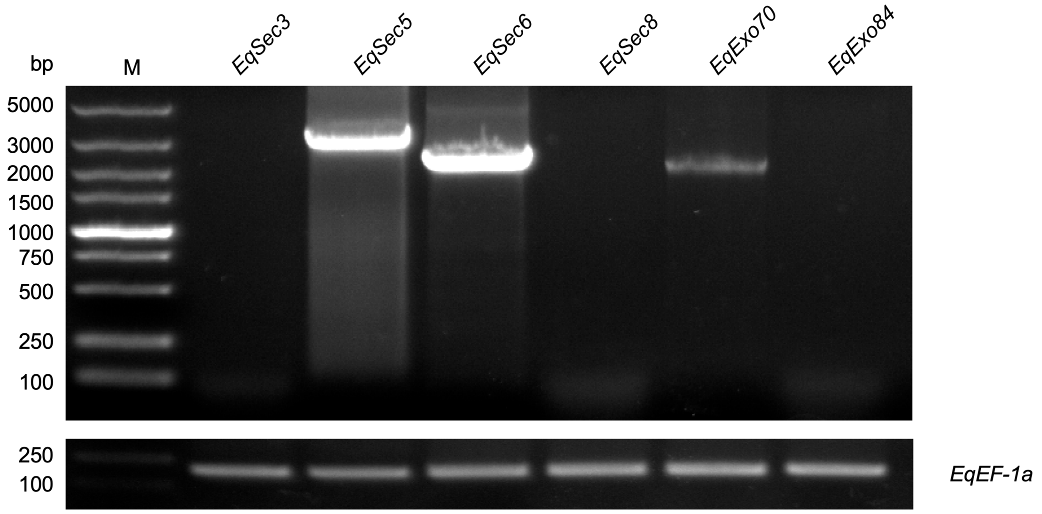

Figure S1 RT-PCR analysis of exocyst subunits using total RNA from *E. quercicola* as a template. *EqEF-1a* was used as the reference control. M, molecular size marker (5 kb ladder).
