# Supplementary material for: The Exocyst Subunits EqSec5 and EqSec6 Promote Powdery Mildew Fungus Growth and Pathogenicity
Source: J Fungi (Basel). 2025 Jan 17;11(1):73. doi: 10.3390/jof11010073 (PMC11767214; doi:10.3390/jof11010073)
Supplement: Supplementary file 1 [file jof-11-00073-s001.zip › Figure S2.pdf]

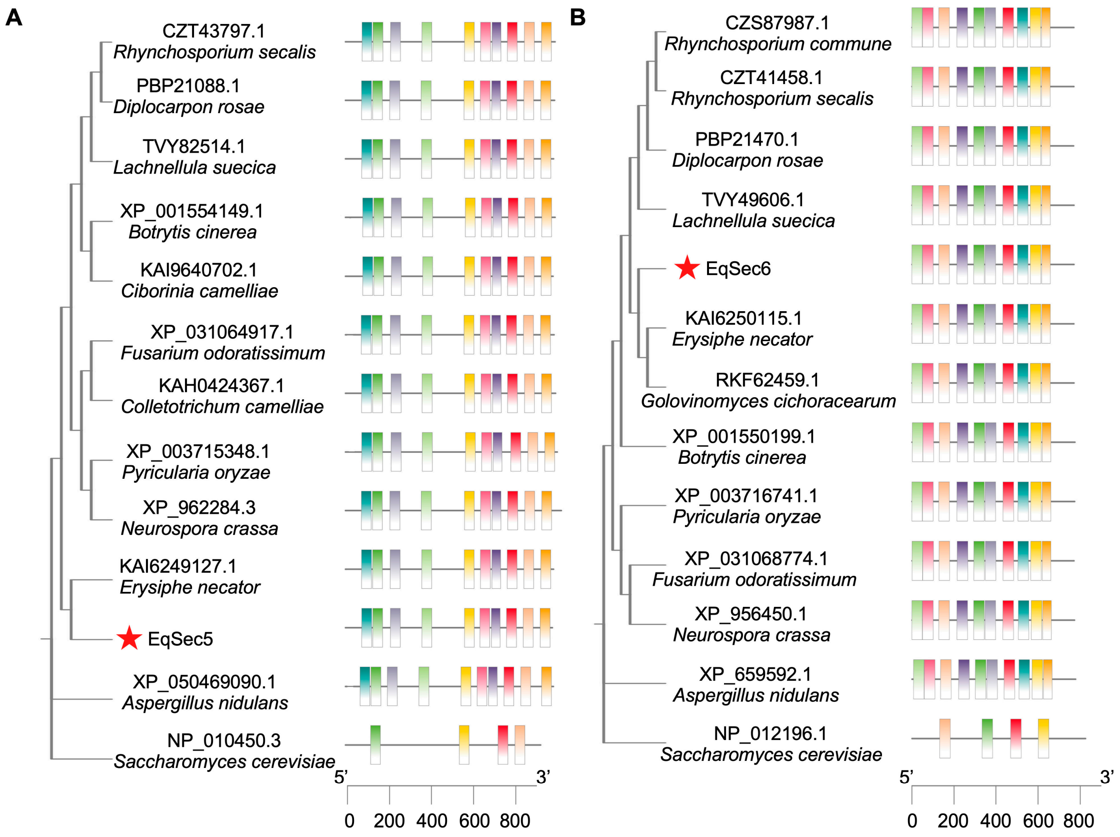

Figure S2 Phylogenetic trees and conservative protein motifs of (A) EqSec5 and (B) EqSec6. Phylogenetic trees were identified using MEGA7. The conserved motifs of the sec5 or sec6 proteins were identified via MEME, shown with different colors representing distinct motifs. The scale bars at the bottom denote the protein sequence lengths.
