# Supplementary material for: The Exocyst Subunits EqSec5 and EqSec6 Promote Powdery Mildew Fungus Growth and Pathogenicity
Source: J Fungi (Basel). 2025 Jan 17;11(1):73. doi: 10.3390/jof11010073 (PMC11767214; doi:10.3390/jof11010073)
Supplement: Supplementary file 1 [file jof-11-00073-s001.zip › Figure S3.pdf]

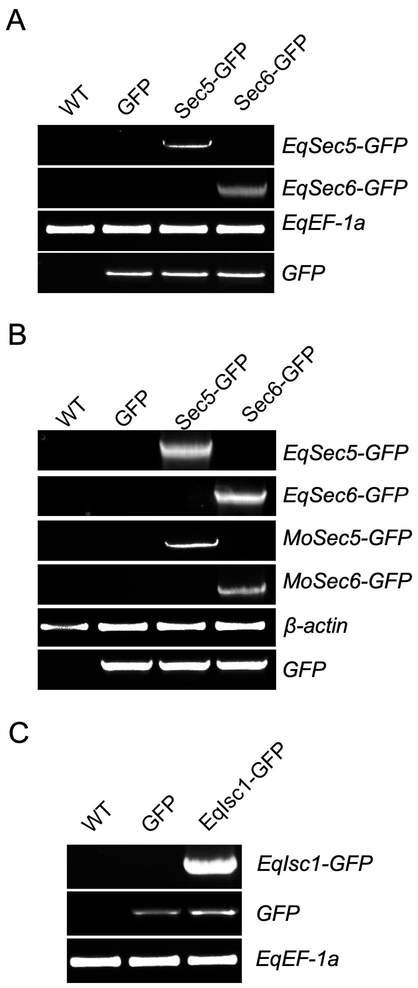

Figure S3 RT-PCR analysis of transgenes in *E. quercicola* and *M. oryzae* transformants. The expressions of GFP-fused exocyst genes in *E. quercicola* (A) and *M. oryzae* (B) were validated. *EqEF-1a* (A) and *β-actin* (B) were used as the reference controls. (C) The expression levels of GFP-fused *EqIsc1* genes in *E. quercicola* were validated. *EqEF-1a* was used as the reference control.
