# Supplementary material for: The Exocyst Subunits EqSec5 and EqSec6 Promote Powdery Mildew Fungus Growth and Pathogenicity
Source: J Fungi (Basel). 2025 Jan 17;11(1):73. doi: 10.3390/jof11010073 (PMC11767214; doi:10.3390/jof11010073)
Supplement: Supplementary file 1 [file jof-11-00073-s001.zip › Figure S4.pdf]

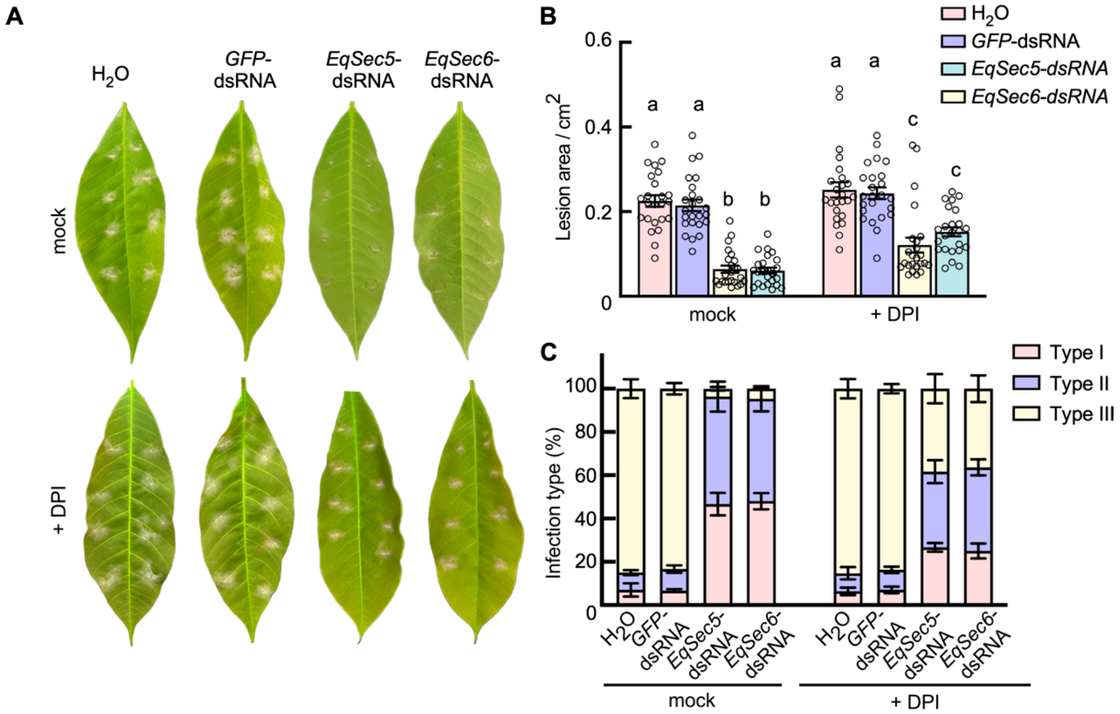

Figure S4 DPI treatment did not fully restore infection by *EqSec5*- and *EqSec6*-silenced strains. (A) Disease symptoms on *H. brasiliensis* leaves with or without DPI treatment. The photographs were captured at 7 dpi. (B) The lesion area on *H. brasiliensis* leaves were calculated at 7 dpi. The data represent means ± SE (n = 24 inoculated sites from 3 independent experiments). Significant differences are indicated by different letters according to one-way analysis of variance and Turkey's multiple-comparison test ($P < 0.05$). (C) The percentage of infection types was calculated at 7 dpi. The infection of each conidium was divided into three types: no penetration (type I), limited hyphal growth (colony radius < 150 μm; type II), and extended hyphae (colony radius > 150 μm; type III). The experiments were conducted three times with similar results.
