# Supplementary material for: The Exocyst Subunits EqSec5 and EqSec6 Promote Powdery Mildew Fungus Growth and Pathogenicity
Source: J Fungi (Basel). 2025 Jan 17;11(1):73. doi: 10.3390/jof11010073 (PMC11767214; doi:10.3390/jof11010073)
Supplement: Supplementary file 1 [file jof-11-00073-s001.zip › Figure S5.pdf]

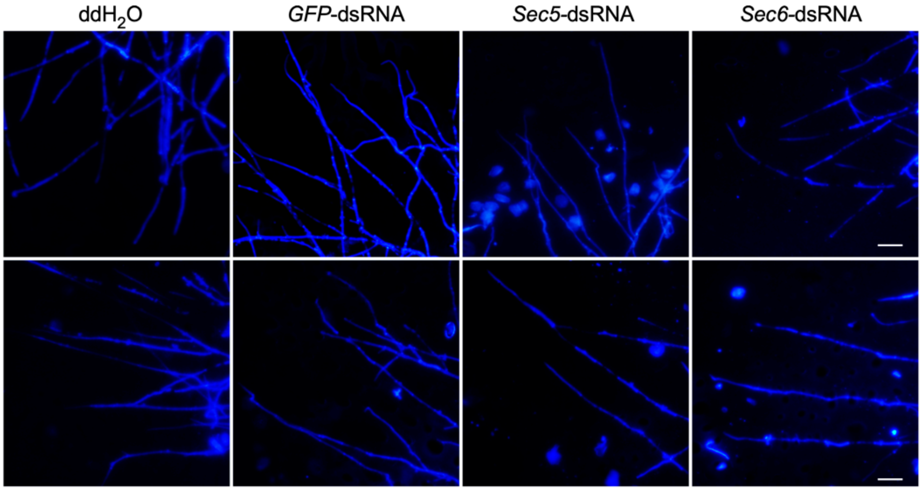


**Figure S5** The *EqSec5*- and *EqSec6*-silenced strains did not exhibit hyphal morphological defects, such as curved or zigzag-shaped hyphae. The strains were inoculated onto the *H. brasiliensis* leaves at 7 days and stained with CFW dye. Bars: 200 μm.
