# Supplementary material for: The Exocyst Subunits EqSec5 and EqSec6 Promote Powdery Mildew Fungus Growth and Pathogenicity
Source: J Fungi (Basel). 2025 Jan 17;11(1):73. doi: 10.3390/jof11010073 (PMC11767214; doi:10.3390/jof11010073)
Supplement: Supplementary file 1 [file jof-11-00073-s001.zip › Table S1.pdf]

Table S1 Primers used in this study.

Application	Forward (5'-3')	Reverse (3'-5')
**Silencing sequence of *EqSec5***	TAATACGACTCACTATAGGGATGAGCGATTATGAATATGA	TAATACGACTCACTATAGGGGTTCGTCTCGTTGTAGTAGA
**Silencing sequence of *EqSec6***	TAATACGACTCACTATAGGGATGGACAGCTCTACGGTCAA	TAATACGACTCACTATAGGGTGTTTTGGGCCTCGGCACAT
**Amplification of *EqSec5* for construction pJNARG-EqSec5RNAi**	GTAGGAACCCAATCTTCAAAGAATTCCCTGTTGCAACACTTTTACG	GAACCACGATTAAATCGAGCCATATGAGCGATTATGAATATGA
**Amplification of *EqSec5* for construction pJNARG-EqSec6RNAi**	GTAGGAACCCAATCTTCAAAGAATTCATAAGTTGTACAGTTCTTTG	GAACCACGATTAAATCGAGCCATATGGACAGCTCTACGGTCAA
**Amplification of *EqSec5* for construction pJNARG-*EqSec5*-GFP**	GTAGGAACCCAATCTTCAAAGAATTCATGAGCGATTATGAATATGA	GAACCACGATTAAATCGAGCCATGAATTCTACTTGTTGCTCATTTGTTA
**Amplification of *EqSec6* for construction pJNARG-*EqSec6*-GFP**	GTAGGAACCCAATCTTCAAAGAATTCATGGACAGCTCTACGGTCAA	GAACCACGATTAAATCGAGCCATGAATTCTTTCACTTTAGACATTATAG
**Amplification of *EqCSEP01276* for construction pJNARG-*EqCSEP01276*-GFP**	GTAGGAACCCAATCTTCAAAGAATTCATGTCTGCCCTTCTCTTCTC	CCACGATTAAATCGAGCCATGAATTCATTCTCATTTGTGTTTTTTG
**Amplification of *EqIsc1* for construction pJNARG-*EqIsc1*-GFP**	ATGACGGAACCATCACCGAG	GCTCCTCGCCCTTGCTCACCAT
**qRT-PCR of *EqSec5***	AAACTGCGCCAAATTTGCCG	AGAAACAATGATGGCGAGAA
**qRT-PCR of *EqSec6***	CACAGTTGCGTAGTGGGCTG	ATCCTTTCGACAAGCTCAAT
**qRT-PCR of *EqCSEP01276***	ATGTCTGCCCTTCTCTTCTC	CTACTGTAAATAATAGTTGA
**qRT-PCR of *EqIsc1***	TTTGACAGCGTGATAAGC	CGGTGTTTCTTTTCGGAAGT
**qRT-PCR of *β-actin***	GGCTCAACCAAATTGACAAG	CTCAGAAAAGACGGATCAAA
**qRT-PCR of *EqCSEP04187***	CTACGAAAGAGGACCGAGTA	GGTTTTCTCCCGCAGCTCTT

**qRT-PCR of *EqEF-1a***	GAACCTTCATCTAACTGC	GTCGTAGTGGTTTGTCAG
**The native promoter of *EqSec5***	AATACTAGTTTTGCCGTCTT	TATTAGATAGAAATTCCTAT
**The native promoter of *EqSec6***	CATAACAAATACCACCCTGT	GCTCTGGAGACTAAAGGATT
