# Supplementary material for: The Exocyst Subunits EqSec5 and EqSec6 Promote Powdery Mildew Fungus Growth and Pathogenicity
Source: J Fungi (Basel). 2025 Jan 17;11(1):73. doi: 10.3390/jof11010073 (PMC11767214; doi:10.3390/jof11010073)
Supplement: Supplementary file 1 [file jof-11-00073-s001.zip › Table S2.pdf]

Table S2 The DNA sequences of *E. quercicola* used in this study

>*EqSec5* cDNA
ATGAGCGATTATGAATATGAGGTGCTTTCACGATATCAATTAAAAACACCATTTCCAACTGAA
TGGCCTGCTGATAAGGATGTGAGTGATGGATCCGATAATGAAGAAAACGAAACTGCGCCAA
ATTTGCCGCAAAAGTCCAAGCCAAGATACTCAGCTCTTGTAAGGGCTGCGGGAAATCGTAA
AAGTGTTGCAACAGGCTCACAGAGAGCTGATAATACATTAGAAAATCTACTACAACGAGAC
GAACCAGACCCACTAGGATCGACTGATAGCGTCGTCGGAAAATTGAAAATTTTGGGTCTAC
CTGTTCAAGACAACATACGACTGCGCAATAAATTTCTCCTCTCATCTACTACATTCTCGCCAT
CATTGTTTCTCTCTCAGGTGCATTCATCAGCCACTACCCAAGATCTTCTTGAAGGCCTAGAA
ATACTGTCCCAGTCAATCGATCAAAAGTCAGCATCGTTGAAAGTGCTGGTGGAGTCTAACT
TTGAACGTTTTGTCAGAGCCAAGGCTACCATTGATAATGTCTACAAGGAGATGAGATATCGT
GGTGAAGAACCAGCACCTACTTTAACACGTCCACGTACTCATTCAAGGCATGCTAGCAGGA
ATAGTTTTCGAACTACTAGTAGTAATCAAGTATCTACACCAACTCATGTTCCCGACATAAAAA
AGAAAAATGCACTCTTAAAGGAAAGTGAGTATGGTGTACTTGGCATCAAGTCTCATCTTCTT
GATGTTTCTGCTAAGGCTGAAGAAGTATGGGGGCCATCTTTAGGAGGACATGAAAAAGAGA
ATAATCTTCGACTTATGACTTCAAGTATGGAACAACTTAAGGAATTATATCAAGTCAGTGGCT
CTTTATCAGAAAGCATTAAACGTAAGGACTATGAATCTATATTAGAGGAATACAATAAGGCA
AAACGATTTGCTAGCGATGCCAAAAATCTCTCTGAAAGACTTAGTACAACTACGGCCACCG
ATTTGCAGATTTTTCAAATATTGCTGACCGCGCACGTTTGGAGTAACGTGTTAGAGATGGTT
GAGGATCTCAAACGTGATATTTGGCGAAAATTATTATCTATTCAAAATAGTTCCTCGAGGAC
AGACACAGGTAGTGAAAATAGACTAGATAAGTACATGGATCATATTGAGATTCTACTTGAAC
TTGGTGTATCAGAAAGTCCTATTTGGGTTTGGCTATCGAATCGCTATAACTCTTTGAAAAGTA
AGATTCAGAGCAGTTCGAGTCAGTACAAGGCAGAGATTGAAATTCTTAGACGGCGACTTGC
CAATTCTGAAAAACCTACATCTCCAGTAATTGCCTCTTCTCATCTGAAATCCTTGAGTCGTC
ACACAACTGAAACTAAGTTGATGAATATTGATTCACCTGGAATTGTCGAATTGTGGGAGAG
AGTTAGTAGTTATGTTAGTATCTTTTTGTCTACTCAAGGCATTTTAGGTGAAATTGTTGAGCT
ATGGCAAGCTATGCAAAGCTTTATTAATGGCCCAGCAAAGAAAAGGCTGATGACTATTTTAA
ACAAAGATTCTCTGGAACATCAGCAACTTTCCGAAAAAGAGATTTTAGAATTACAAAAAGG
AATTCATGAACTTGTTAATATGATTCGAGAGAGTATTTCTGAACTTTTTTTCAATGAGCCAGT
CGATGACATATCGTCTCTTCTCTCACCCCTGCTACTTACACCGCGAACTCCTGCATTTCCTTC
TCGGCTAAGTCCTACTTTAAATGCTTTTCGAGACTTTAATGCAAATCAAGATTCTAGTATTTT
ACCGCCTCCATCTATAAAAAGAGGTGAAGCATGGGAAAAGTTTGCATTTTGGCCTCCTTGG
TCTAATTCTTTGAGTGCTGTCCACTACCTTGCGAAAGTGTTGGTTCATATTGGCTCGGGAGC
AAACGAGATGGCTGCAGTTTTCTCAGAAGAACAGGAATTCGATATATCTAAAGAGCAATTG
AAATCTTTCGTAAGTGGTATTCGAGAACGTTGTGTAAATGCTTTTATTGTGGCTTGGAATAA
AGATGCCGAAGATATGAAGGTGCTAGAGGACTGGAGAAGATCGCCCGAAAATCATAACTTA
ACAAATATGCCAATGTACTTTGGTTTATTTGGGAATATCATCTTGAGTGGTATGCAGAAGATC
TTATACATACCAGAGGCCTCAACCAAAACAGACTCGGTGAATGTAGTCTCACCACCTCCTG
CCAAGTTACTGCAAGTGGTTCGAAGTCAATTTGTGTCAACTTTATATAAGTCCCTCGCTGGA
TTGGTAGAAAACGCCGAACAGTCTGTTACTAAGTCTGATGATGTGTGGACTCCAGACATTG
ATAGTCTTGCAAGCTCCACGCAAATGGTAAATGCGATCAATTTGGACGTAGGGACGGTTAA
CTCAAGAGAACGGAATGTCCGCATGCTTATAACTCTCAGTAATATGCAGGCTCTCTTCATTG
ATGTGGTGCCGAACATGGTCGCCCAGTTCGAGAGTGCCTTTGCTGTAAAGTTGACAGATGA
AACTAATATCATTCGAGATATTCTTAATCAGATTGATGCTCGTCTTTTTTCCTCTTTCACTCAA

CCTTATGTTGAAACCCTTCGTAACAACATTCGTGTCGGTATTACTTCATCAATGCAGACTTTA
ATCGAGAAGCCGAGAGACGTACGTCCTTATGTCTATGAAGTCTTATTATCATTAGTCCTTCTA
CATAGTGAGGTCTCTACTACCGCTGCTTCTCTTACTGTGAAAATTCTATCATATCTTTTGGAG
CAAATATCAGAAGAGCTTCTTGAATCATTGAAACTTCGCTCACAATGGTCCCTTCCGGATCT
CATGCAAGCAACACTTGATGTTGAATTTATGTCACAAACTTTAATGCAATACAGTACTGACA
GCGCTAGCGAAATTCAAGGTCTAATTTATCAAGAGTTGGATAAAGGGACAGATAATACTTCT
AGGGCTGAACTCCAGACTGAGTTACCAGAAATGAGGACCGTACTAAAGAGGCTTCGAGAT
AAAAGCCGGAGTGAATTTGTTTGCTTTAAAAAGAGTAGAAGTCGAACCGAAAACTCTCATG
CATCAAGCTTAACAAATGAGCAACAAGTATAA

>*EqSec6* cDNA
ATGGACAGCTCTACGGTCAAGCTTACAGAGATTTTGAAATATCCTGATGATATAGACAAGGT
TTCGGCTTTGAAACTCGAATATGTCCGAAAGAAAGCTGCGGTCGATTCACAGTTGCGTAGT
GGGCTGAAGGAACAATCAGAAATTACACAATCAGGAATGAGTCGAATAAATGATGGCCAAA
GAACTGTACAACTTATTAAAGATGAGATGATGAGAGTTGACAAACTATGTGCCGAGGCCCA
AAACATGATCCGAGATTTCCCTAATATCAATCTTGTTTCACAAACACATAAGAATTTCATAGC
TGTACAAAAAATGAGAAATGATTTGCAAACATTCAATGACCGCATTGAGCTTGTCGAAAGG
ATGCTGAGGGAGGATGATAGAGATCCTGAGAATATGCCTAACCTTTTGCCAGTACATTATGA
GCTTACCCAACTGAGAAATATAAGGGATAGCGCTATGGAACAAGTTGCACGGGCGGATGAT
CAAAGCCTTCAAAATACTTTAGAAGACTATTTCGTTAAGCAAGATGAAGTTATAGAATGGTT
TGACGAGCATATCGGCATGATTGCACTAGATATCATAAATGTTCTCATCAACGGCAATACAAG
TATGATAGTAAGATTTGCAGTAATTGTAGAAGCTGAAGAAAGGAGTGATAAGAGAATAAAA
GCGATGCAGGAGGCATTGAAAGATCATAAAGAAATGGCCAGTAGGTTTCAAAGCATCACAG
ATGGTGCTAGGACCGTAAAAGGTTATAAAGAAAAATTTTTAATGGCTATAAAACTTCATGCT
GATGAGCAGATGAGTGCTACAAAAGAATCTTTTTTACAGGATCCAGGACGGTTGGAGAAAA
GTTTGAAATGGTTTTTTAATGATTTAAATGCCATAAAGCAAGGTATGGTCCCTCTCATGCCCA
AAAAATGGAAGATTTTCCAGACATATGGCGAAATTTATCACAAAATTATGCATGACTTTCTCA
TTGGTATGGTTGATGATCCCGATACTAGCTCGGCCAATACATTATCTATTCTGAACTGGCCGG
AAAAATATTACGCAAAAATGGGAAGGCTAGGCTTTAACAAGTCCGAGTTACGTCCTCATGT
ACTAGATGAACGAGAAAATGAGCTAGTTAGGGACTTTCAGCAACTTATCATTCGCTTCCTAG
ATCAGTGGCTGGATCGGATCTTCAAAACTGAACAAAAAGATTTTACAGATCGAAATGTTGAT
GGAGGTAATCTTAACACAGATGAATACGGTTATTTTCGAACAAAAAACCTCGTCGACATGTG
GAGAATGCTCGCTGAGCAGATAGAGGCGGCCGAGAACTCAAAGCGAGTTGATGTATTAGA
GGGCGTGATTGATGCGATGATTTTCCGACTTCAGACTCGGCAAAGAAATTGGCAGAAAATG
TTAGATGATGAAGCTTCCAAATATTTTGGGAACAATCCTGAGTCTGAAGGATTTCAAGCTTT
ACAAGACTGGCTTGTGGCTACTGCTAATGATCAAATCGCATGCATAGATGATAATGAAGAGG
AGGAGCGATTTGCTTATGTGACTATATTTCGACAACAATTTGAGCCACTTGTAACCCCCTCTT
ATTTAGAGCGCGTTGAAGATAAGCTCAGTATACTACGAGATGGCTACGTGGATCTTAGCACA
CACTGCGTCGCTAAATTTGGGCAGCTCATTTTTGCTGTAGATTTCCGGACAGTTCTACCGGA
TTTTTTTACACCCAAATGGTACACGGCCAATGCTATGAAGCAGATCGTTGTGACTTTTGAAG
AATATATTGGAGACTACAAAGCTGTTCTGCATTATTCACTTTTAGATATCTTTATAGAAGAACT
TGCTGATGAGCTTCTCATTCGTTATCTATCAAGTGTAAAAAACAAGGGAGCTAAATTTAAAC
GTCAAGATCCATTTAAGGAAAAAATTTTCAGTGATGTATCTGTTGCATTTGAGTTTTTTAACA
ATGGCTTTTTAAGTCCTGATGTAGCTGATGTGATTAAGCAGAAATGGAGAGTTACAGAGAGC

TTTTTAGCCCTACTTGAAACGGAAAAGAGTTTGTTACCTGATATATTTGCAAGCTTTAAAAC<br>AGAGTACTGGGATCTACAATTCAGCTGGGTTGAAGCTGTGCTGAGATCAAGAGATGATTTT<br>GATAGAAGCCTACTAAATGCCGTGAAGTCTAAAGCTGCTCAGATAAATGTCCCTAGGGGAAT<br>AGAAACTATAATGTCTAAAGTGAAATAA
>*EqSEC5 silencing sequence*<br>GTTCGTCTCGTTGTAGTAGATTTTCTAATGTATTATCAGCTCTCTGTGAGCCTGTTGCAA<br>CACTTTTACGATTTCCCGCAGCCCTTACAAGAGCTGAGTATCTTGGCTTGGACTTTTGCG<br>GCAAATTTGGCGCAGTTTCGTTTTCTTCATTATCGGATCCATCACTCACATCCTTATCAG<br>CAGGCCATTCAGTTGGAAATGGTGTTTTTAATTGATATCGTGAAAGCACCTCATATTCAT<br>AATCGCTCAT
>*EqSEC6 silencing sequence*<br>ATGGACAGCTCTACGGTCAAGCTTACAGAGATTTTGAAATATCCTGATGATATAGACAAGGT<br>TTCGGCTTTGAAACTCGAATATGTCCGAAAGAAAGCTGCGGTCGATTCACAGTTGCGTAGT<br>GGGCTGAAGGAACAATCAGAAATTACACAATCAGGAATGAGTCGAATAAATGATGGCCAAA<br>GAACTGTACAACTTATTAAAGATGAGATGATGAGAGTTGACAAACTATGTGCCGAGGCCCA<br>AAACA
>the native promoter of *EqSec5*<br>AATACTAGTTTTGCCGTCTTTTAACTGTGGGGTCACATCAACAGGCTCCCATTTGATAGG<br>CGCCTAAGTTACGTCAAAAAATGACAGCTTTTATTTCGTTGAAGAACAAGCCTTTGCAGC<br>CTCAAAAATGTCTTTCACTGACTGAGAAATCTCAGGACCAATCCCATCACCCTCTATTAA<br>GCTGACAGCAAAAAGACCCTAGTTGAAAGTTAGATAATTCTGTATATCTGTGCCTTAATG<br>TATGGCATAGATGTGCAATAACAAGTACATTTTCGTCTTTCTGACCATGAAATCCGGCAA<br>CTTTCTCAGATGTAGTATTGGCATAAGCTCTTCGGATCTAAGTCTCAAAAGTATCAGCAA<br>AATAAATTCATTTTAGCGCTTCGTTTTTACAGACCTGAGAGGATATCGATGCTAGATTTT<br>GAAATCGAGGGGAAAAGCACTGCCGCCTAATAGGCACAGATAAACGGTACATATCAACTG<br>ATTATATTATTTCAACCTATAATTTTTTGTCTAAAATAATACTTCTTGAATGGAATGGTT<br>GATGTGTATATTCAGATTGGTATATTTGGCCCGAGAGCACTTAGCGCTTATACGACAATC<br>CTACATGTTGATTGATATTTGTCTAAATTACCTTCTTCCAGAATCGAATCCTACCCCACC<br>AAAACGAATCCTCCCTGCGCCAAACAAATTATGAGGAAGTTTTTTACACATTACTACAAT<br>TATATCTAATTTGTTTATGCAGATGGACGAATTTATGAATTTGAACCTCAACTCTATGGT<br>ATAGGAATTTCTATCTAATA
>the native promoter of *EqSec6*<br>CATAACAAATACCACCCTGTTTATTAGCTTTATCTTGCCTCACATTCAAGATTTGTACAAACT<br>TACTTCAAAGTTTTAAAAAATTGGGTAGCAGTTAATGTCTGCTATTGATAGTATAATGAGCCA<br>CGTAATATCAACGTAAAAGAACGAGAAATTAATTTAGAGTACAAGTAAGAAATCTAAGACTT<br>TATTCTATTTTATTTGGTTGGAGAAAACTTGATAGCTTTTGATTTTACGATGTAATCTTGTTTA<br>TTCACCTCATTTTTATCGTGGTACTGATTTTTCAATATAAAATCCTGGGCTAGGTCTCCTTTCA<br>GCTTTTAAATCATCTTTGGTCAATTCTTTTTCAACTTCTGTCACTGAAACTAGCTCTTTAACC<br>CGCATGATTTGACCAGCAGTCTCAGGAGAGACCGGAAGAAAAATGGTTTCATCCGTTTTC<br>GAAGGCCAAGCGCCTTAAGTACTCCTCTGGAACGTTTTGTTAAGCCAATACCGGATCGCATA<br>AGCGTTATTCGAAAGAATGCCATTTAATCTTGGAGAATTAGCCAATCTTTCAAATTTACTCTT<br>AAATTAAATGGCCACCTAATATTTTTAATTGTGCTAAAGTTGCTATTGGTTTCGTGATTTGTG

ATTGAATCAGGAATAATGTTTATTAGTCTCTCAATCACATGTTTTATCATCTCCACAAGAATAC
TTGAAAACACTATCGGGGCGGAATAACAAATATTCCCTCCACTTGTCTTGAAAAATTACTTG
TTGATAGATATATAGGCTCAACTCCAATCCTTTAGTCTCCAGAGC
